# Direct microscopy versus sputum cytology analysis and bleach sedimentation for diagnosis of tuberculosis: a prospective diagnostic study

**DOI:** 10.1186/1471-2334-10-276

**Published:** 2010-09-21

**Authors:** Pamela Hepple, Pascal Nguele, Jane Greig, Maryline Bonnet, Vinciane Sizaire

**Affiliations:** 1 Manson Unit, Médecins Sans Frontières UK, 67-74 Saffron Hill, London EC1N 8QX, UK; 2 Mindouli Hospital, Pool Region, Republic of Congo; 3 Epicentre, 53-55 rue Crozatier, 75012 Paris, France

## Abstract

**Background:**

Diagnostic options for pulmonary tuberculosis in resource-poor settings are commonly limited to smear microscopy. We investigated whether bleach concentration by sedimentation and sputum cytology analysis (SCA) increased the positivity rate of smear microscopy for smear-positive tuberculosis.

**Methods:**

We did a prospective diagnostic study in a Médecins Sans Frontières-supported hospital in Mindouli, Republic of Congo. Three sputum samples were obtained from 280 consecutive pulmonary tuberculosis suspects, and were processed according to WHO guidelines for direct smear microscopy. The remainder of each sputum sample was homogenised with 2.6% bleach, sedimented overnight, smeared, and examined blinded to the direct smear result for acid-fast bacilli (AFB). All direct smears were assessed for quality by SCA. If a patient produced fewer than three good-quality sputum samples, further samples were requested. Sediment smear examination was performed independently of SCA result on the corresponding direct smear. Positivity rates were compared using McNemar's test.

**Results:**

Excluding SCA, 43.2% of all patients were diagnosed as positive on direct microscopy of up to three samples. 47.9% were diagnosed on sediment microscopy, with 48.2% being diagnosed on direct microscopy, sediment microscopy, or both. The positivity rate increased from 43.2% to 47.9% with a case definition of one positive smear (≥1 AFB/100 high power fields) of three, and from 42.1% to 43.9% with two positive smears. SCA resulted in 87.9% of patients producing at least two good-quality sputum samples, with 75.7% producing three or more. Using a case definition of one positive smear, the incremental yield of bleach sedimentation was 14/121, or 11.6% (95% CI 6.5-18.6, p = 0.001) and in combination with SCA was 15/121, or 12.4% (95% CI 7.1-19.6, p = 0.002). Incremental yields with two positive smears were 5/118, or 4.2% (95% CI 1.4-9.6, p = 0.062) and 7/118, or 5.9% (95% CI 2.4-11.8, p = 0.016), respectively.

**Conclusions:**

The combination of bleach sedimentation and SCA resulted in significantly increased microscopy positivity rates with a case definition of either one or two positive smears. Implementation of bleach sedimentation led to a significant increase in the diagnosis of smear-positive patients. Implementation of SCA did not result in significantly increased diagnosis of tuberculosis, but did result in improved sample quality. Requesting extra sputum samples based on SCA results, combined with bleach sedimentation, could significantly increase the detection of smear-positive patients if routinely implemented in resource-limited settings where gold standard techniques are not available. We recommend that a pilot phase is undertaken before routine implementation to determine the impact in a particular context.

## Background

Tuberculosis (TB) is a major public health problem, with an estimated 2 billion people infected with tubercule bacilli worldwide. Estimated global prevalence of the disease is 139 per 100,000 population [1]. In recent years, the prevalence of TB has increased steeply, driven in large part by the HIV epidemic. TB in patients co-infected with HIV can be difficult to diagnose, especially by sputum-smear microscopy--often the only diagnostic tool available in resource-limited settings. The medical humanitarian aid agency Médecins Sans Frontières (MSF) works in settings where the health infrastructure needed to treat patients with TB, many of whom are co-infected with HIV, is either absent or extremely limited.

The gold standard for diagnosing pulmonary tuberculosis is culture of sputum on Löwenstein-Jensen medium. However, due to lack of access to culture facilities and the long turn-around times involved with sputum culture, most programmes use direct Ziehl-Neelsen microscopy for detection of acid-fast bacilli (AFB) in sputum smears. This technique, when performed optimally, has reported sensitivities ranging from 61.8% to 70% compared with the gold-standard [2-4]. Sensitivity is reduced if samples are of poor quality, which is often the case in children and in people who are HIV-positive, since sputum expectoration is more difficult [3,5].

Sputum samples are also often inadequate in patients who are immune-compromised or who have not been given correct instructions on sputum expectoration [6]. Patients frequently provide pure saliva or very small amounts of sputum in saliva instead of an appropriate, purulent, sputum sample [7]. A negative result issued on examination of such a sample is misleading, since it implies that a correct sputum specimen has been examined. Sputum cytology analysis (SCA), in which smears are examined and graded according to an algorithm, has been proposed as a means to ensure sufficient sample quality. Further samples can be then requested if quality is inadequate [8].

Sputum concentration by homogenisation and microscopic examination of the sediment can increase the detection rate of AFB. Several concentration techniques using sedimentation or centrifugation have been reported [9-11]. Bleach homogenisation followed by overnight sedimentation can significantly increase detection of AFB compared with direct microscopy [12-14]. A systematic review of this technique found an increase in sensitivity of 33% in one of four studies where culture was used as a gold standard, and a mean incremental yield of 6% in four studies in which culture was not used [9]. Centrifugation resulted in higher yields than sedimentation. Cattamanchi and colleagues [15] found that bleach concentration resulted in small increases in sensitivity and small decreases in specificity. However, centrifugation requires electricity as well as equipment that might not be present in peripheral laboratories in resource-limited settings. Homogenisation can be achieved with various agents, such as sodium hydroxide (NaOH) and N-acetyl-L cysteine (NALC) [11]. Household bleach--sodium hypochlorite (NaOCl)--can also be used, and it has the advantage of being easily available worldwide.

Due to the variability of results to date, it has been recommended that further research is done before the adoption of bleach concentration as a standard diagnostic method [10].

We did a prospective field assessment in routine conditions in a peripheral laboratory in an MSF-supported hospital in Mindouli, Republic of Congo. We aimed to compare the proportion of smear-positive patients detected with and without bleach concentration of sputum by overnight sedimentation with validation of all samples by SCA. Our hypothesis was that the combination of SCA and bleach sedimentation would significantly improve the detection rate for smear microscopy of pulmonary TB.

## Methods

### Setting and patients

The study was conducted between October 2006 and March 2008 in Mindouli, Pool Region, Republic of Congo. MSF was supporting the district Ministry of Health hospital, with services ranging from outpatient care, maternal care, and treatment of infectious diseases such as tuberculosis and HIV/AIDS, to psychosocial counselling and emergency surgery. Fighting between rebels and government forces meant that health care services had been neglected in the region, and food insecurity had resulted in high rates of malnutrition. After a ceasefire, internally displaced people returned to the region, leading MSF to start interventions in Ministry of Health hospitals in Kinkala, Kindamba, and Mindouli. Adult HIV prevalence is less than 4%, with a TB prevalence of 449 per 100,000 population [16,17]. Patients were included according to the following criteria: 15 years of age or older; suspected pulmonary TB with cough of at least 3 weeks duration; and non-ingestion of anti-TB drugs in the previous month. Every patient suspected of having pulmonary TB was assessed by a TB nurse and invited to participate in the study. An informed consent form was signed by those who agreed to participate. All patients invited to participate agreed to do so. Patients were accommodated in the hospital until a final result was available, according to standard procedures in the project setting.

### Ethics approval

Ethics approval for the study was obtained from the MSF Ethics Review Board. The study was also approved by the Ministry of Health Provincial Directorate of the National TB Programme of the Republic of Congo.

### Specimen collection and processing

Patients were given instructions on sputum production. Collection of the first sample was supervised by a TB nurse. Samples lacking any purulent material were rejected and the patient asked to try again. Patients were given a sputum container for expectoration at home the following morning. When the second sample was brought to the laboratory, it was examined macroscopically. If no purulent material was present, a laboratory technician supervised collection of a replacement sample. A third container was provided for expectoration the following morning. After processing, if there were fewer than three good sputum samples the patient was asked to provide further spot samples.

Results were passed on to the clinician once all requested samples had been processed for direct microscopy and independently of results of sediment microscopy. For the case definition of one positive sample, any positive sample in the sample batch was considered eligible. For the case definition of two positive samples, any two positive samples in the sample batch were considered eligible. Direct microscopy smears were processed according to the standard hot Ziehl-Neelsen staining procedure and examined for SCA and presence of AFB.

After direct smears had been made, the remainder of each sample was processed for bleach sedimentation according to the procedure outlined by Bonnet and colleagues [11], with the exception that samples were manually shaken for 30 seconds instead of being vortexed. In brief, a direct smear was made from each sputum sample. An equal amount of household bleach was then added to the sputum sample in a screw-cap tube, shaken for 30 seconds, and allowed to sediment overnight. The following morning, the supernatant was decanted and a smear was made of the sediment. Samples were then processed according to the standard hot Ziehl-Neelsen staining procedure, and the smears were examined for presence of AFB.

Both direct and sediment smears were examined in a blinded manner by two laboratory technicians, and graded according to the Pasteur scale, which was then converted to the WHO-IUATLD scale. In case of discordant results, both readers repeated the slide reading until a consensus was reached. Monthly blinded quality control (QC) was done by the laboratory supervisor using five positive and five negative randomly selected smears from both direct and sediment subsets. Direct slides were also assessed for sputum cytology analysis agreement. External blinded QC was done halfway through and at the end of the study on 10% of positive and 10% of negative samples in both subsets. QC for SCA was also done on 10% of direct smears.

### NaOCl solution

"Lacroix" bleach was purchased from a supermarket in Brazzaville, Republic of Congo. Enough was purchased for the study duration. The stated chlorine concentration was 2.6%. To prevent reduction of chlorine activity from repeated exposure to air [18], each 2.5 L bottle was decanted on opening into 25 mL brown glass bottles for daily use. Each new 2.5 L bottle was tested for residual chlorine availability using the Palintest (R) diethyl-p-phenylene diamine (DPD) pool testing system, with the bleach in a 1:10,000 dilution. The bleach was always in the range of 1-1.5 mg/L at this dilution. A small bottle was randomly selected each week for testing with DPD, at the same dilution, and was always between 1-1.5 mg/L, indicating no reduction of activity. A Ziehl-Neelsen smear was made from each new bottle to ensure no contamination with mycobacteria.

### Sputum cytology analysis

Smears were examined with ×10 objective (×100 magnification) and categorised according to the SCA algorithm (Table 1).

**Table 1:** Sputum cytology analysis definitions [8]

	Definition
Saliva	Presence of squamous epithelial cells and/or dark patches; absence of particulate material, absence of significant number of polymorphoneutrophils/macrophages
Mucus	Acellular, defined edges
Degraded sputum	Presence of dark patches of material on ×10 objective which, using ×100 objective, are totally covered with blue-staining non-mycobacterial bacteria; presence of polymorphoneutrophils/macrophages and/or fibrils
Insufficient sputum (good quality, insufficient quantity)	Presence of patches of polymorphoneutrophils/macrophages and/or fibrils, but filling fewer than ten fields when using ×10 objective
"Good/real" sputum (good quality, good quantity)	Presence of ten or more fields of polymorphoneutrophils/macrophages and/or fibrils when using ×10 objective

### Sample size

For a power of 80% at 0.05 precision, and an expected increase in positivity from 27% to 32% with SCA and bleach sedimentation, the sample size required was 543 patients (McNemar test).

### Data analysis

Data were double-entered in Excel 2000, cross-checked, and analysed using Excel 2000 and STATA v10 (College Station, Texas, USA). The exact McNemar's test was used to compare matched data for direct versus sediment results, using a case definition of either one or two positive results. A positive result was defined as ≥1 AFB per 100 high-power field. Analysis was done on the outcome based on obtaining one or two positive samples to reflect a change in the 2007 WHO recommendations that occurred halfway through the study [19]. We analysed the smear-positive detection rate of several combinations of direct and bleach sedimentation microscopy to identify the optimal algorithm for detection of smear positives, with a case definition of one positive smear and using two samples. An alpha of 0.05 was considered significant, and exact binomial 95% CIs were calculated.

## Results

Patient enrolment progressed more slowly than expected, with only 280 patients enrolled after an extension of the study from 1 year to 18 months. The total number of samples obtained was 890. Of the 280 patients enrolled, 41% (115) were male. The mean age was 35 years (SD 12.1). Nine patients (3.2%) were lost to follow up, and three patients died.

Most patients (223/280) provided three samples. 49/280 provided four or more and 8/280 provided fewer than three.

Table 2 shows the detection rates of the different techniques and the incremental yield of bleach sedimentation and SCA over direct microscopy alone. Using bleach sedimentation and a case definition of one positive smear, 14 extra patients were detected, which was 10.4% (95% CI 5.8-16.8) of all positive patients detected (without SCA); this was an incremental yield of 14/121, or 11.6% (95% CI 6.5-18.6) (Table 2). One patient was positive on direct microscopy and negative on sediment microscopy, resulting in a total of 121 patients diagnosed by direct microscopy, of which 120 were positive on sediment microscopy. The incremental increase of sediment microscopy only was 10.7% (95% CI 5.8-17.7), meaning there would have been fewer patients (1) detected if direct microscopy had not been used. The combined use of bleach sedimentation and SCA gave an incremental yield of 15/121, or 12.4% (95% CI 7.1-19.6; Table 2).

**Table 2:** Detection rate of different techniques and comparison of incremental yield over direct microscopy alone

	Case definition of one smear		Case definition of two smears	
	N detected (% of all patients)	Incremental yield over D alone: N (%; 95% CI)	N detected (% of all patients)	Incremental yield over D alone: N (%; 95% CI)
D	121 (43.2%)*	-	118 (42.1%)	-
D+SCA	122 (43.6%)	1 (0.8%; 0-4.5)	118 (42.1%)	0
B	134 (47.9%)†	14 (11.6%; 6.5-18.6)	123 (43.9%)	5 (4.2%; 1.4-9.6)
B+SCA	135 (48.6%)‡	15(12.4%; 7.1-19.6)	125 (44.6%)	7 (5.9%; 2.4-11.8)

D = direct microscopy. SCA = sputum cytology analysis. B = bleach sedimentation. *One patient was positive on direct microscopy and negative on sediment microscopy, resulting in a total of 121 patients diagnosed by direct microscopy, of which 120 were positive on sediment microscopy. †One patient positive on direct but negative on sediment in three samples (without SCA). ‡One patient positive on sediment but not on direct microscopy after SCA.

When a case definition of two positive smears was used, bleach sedimentation gave an incremental yield of 5/118, or 4.2% (95% CI 1.4-9.6; Table 2). Only five extra patients were detected--4.1% (95% CI 1.3-9.2) of all patients detected as positive with this case definition. After SCA, two extra patients were detected on sedimentation but none on direct microscopy. The incremental yield of both techniques was 7/118, or 5.9% (95% CI 2.4-11.8; Table 2).

Results were analysed to determine the optimal algorithm for detection of smear positives, with a case definition of one positive smear and using two samples (Table 3). Sedimentation of both sample 1 and 2 would have resulted in the highest positivity rate (46.8%). A direct smear from the first sample, followed by sedimentation of the second, was almost as effective (45.4%). The incremental case detection of B1+B2 (sedimentation of first and second sample) compared with D1+D2 (direct smears for both) would have been 11/120 (9.2%).

**Table 3:** Smear-positive detection rates of direct and bleach smear microscopy (excluding SCA)*

Approach	Smear-positive detection rate			Compared with D1+D2
	n	%	95% CI	p
D1+D2	120	**42.9**	37.0-48.9	-
B1	125	**44.6**	38.7-50.7	0.23
B1+B2	131	**46.8**	40.8-52.8	<0.01
D1+B1	128	**45.7**	39.8-51.7	0.01
B1+D2	127	**45.4**	39.4-51.4	0.04
D1+B2	127	**45.4**	39.4-51.4	0.02
D1+B1+D2	128	**45.7**	39.8-51.7	0.01
D1+D2+B2	127	**45.4**	39.4-51.4	0.02
D1+B1+B2	132	**47.1**	41.2-53.2	<0.01
B1+D2+B2	127	**45.4**	39.4-51.4	0.02

D = direct. B = bleach sedimentation. *Based on the 2007 WHO smear-positive case definition (one smear positive with ≥1 AFB/100 high-power field).

83% (737/890) of samples, including extra samples requested after SCA, were good-quality sputum. 15% (137/890) were insufficient or degraded sputum. 2% (16/890) of samples were saliva or mucus. One patient failed to produce any sputum samples, but was retained in the study (three saliva samples, negative on direct, all positive on sediment).

All poor-quality samples (degraded, mucus, and saliva) were negative on direct microscopy. Using bleach sedimentation on 47 poor-quality samples resulted in seven positive results (18.4%: four [10.5%] for degraded, three [7.9%] for saliva), which improved the overall detection rate (p = 0.0215). The proportion of good-quality samples giving a positive result was significantly more using sedimentation (360 of 890) than direct microscopy (347 of 890; p = 0.0044).

Table 4 shows the number of patients able to produce good-quality sputum samples before and after SCA. Nine patients were able to provide at least two good samples after SCA (3.8%; 95% CI 2.8-7.1) and 17 patients provided three or more (10.8%; 95% CI 6.8-16.0). Of 68 patients unable to produce three or more good-quality samples, 30 (44.1%) could not produce further samples, despite repeated efforts; three (4.4%) died before further samples could be requested; nine (13.2%) were lost to follow-up; and data for 26 patients (38.2%) are missing.

**Table 4:** Number of patients able to produce good-quality sputum samples before and after SCA

	Able to produce three samples (n [%])	Able to produce two or more samples (n [%])	Unable to produce any samples (n [%])
Before SCA	195 (69.6%)	237 (84.6%)	12 (4.3%)
After SCA	212 (75.7%)	246 (87.9%)	9 (3.2%)

SCA = sputum cytology analysis.

### Quality control

A total of 1780 slides were read. Fewer than ten inter-reader discrepancies were noted and all were resolved upon re-reading. External QC results showed excellent agreement for direct and bleach sedimentation techniques (both 99%), with very good agreement for SCA (90%).

## Discussion

Our results show that bleach sedimentation and SCA increased the detection of smear-positive TB patients by 12.4%, which is in line with the findings of other studies reporting incremental yields ranging from 7% to 253% [20]. A meta-analysis found that, in studies comparing overnight sedimentation with culture, the mean increase in sensitivity was 23%, whereas in those without culture, the mean incremental yield was 5% [9].

The main implication of our study is that implementation of bleach sedimentation would significantly increase the detection of smear-positive patients if routinely implemented in resource-limited settings where gold standard techniques are not available.

SCA is a low-workload intervention which takes roughly 15-30 seconds to perform, and so can easily be integrated into routine examination. Our QC results show that the technique is highly reproducible. However, bleach sedimentation increased workload and delayed results by 1 day compared with direct microscopy.

The combination which yielded the highest positivity (45.4%), using a maximum of two samples and incurring minimal extra workload and delay, was a direct smear for the first sample and bleach sedimentation for the second. This approach allows rapid identification of patients positive on the first direct smear without waiting for the results of sedimentation. The use of two samples (the supervised sample and the one obtained the next morning) increased sensitivity by allowing for one sample to be of poor quality, and by concentrating the sample which is likely to be of the best quality (the morning sample). This procedure is based on the 2007 WHO recommendations [19,21].

One patient detected using direct smear microscopy was negative on sediment smear. This result could have been caused by chance, with the only AFB-containing portion of the sputum being used to make the direct smear.

The analyses of residual chlorine activity showed that there was no reduction of chlorine activity after the bleach was decanted into small bottles. Because homogenisation activity is also visible microscopically, (i.e. lack of homogenisation results in intact white blood cells after sedimentation), it should not be necessary to measure residual chlorine activity routinely.

Sediment smears were fragile and easily washed off the slides during staining. Attempts to prevent this with the addition of bovine serum albumin made smears difficult to decolourise, and the practice was discontinued. AFB in sediment smears were also found to fade; we therefore recommend that QC is performed within 6 months of staining.

Our results showed that poor-quality samples are more likely to be negative on direct and sediment microscopy than good-quality samples, and that both types are significantly more likely to be positive after bleach sedimentation than on direct microscopy. 10.5% of degraded samples were negative in direct microscopy and positive on sedimentation. Although the sample size was small (4 of 38), this result suggests a need for further investigation. With the three saliva samples that became positive after sedimentation, a small amount of sputum was probably present.

A recent study has stated that macroscopic evaluation of sputum for quality is as effective as microscopic techniques such as SCA [22]. The study highlighted that rejection of samples based on cytological criteria would have resulted in some positive samples being missed. However, SCA focuses on the use of cytological classification to request extra samples when negative samples are of poor quality, and all smears are examined for AFB irrespective of quality. Although the actual number of increased smear-positive patients detected with SCA was low in our study, it can be inferred that the negative results obtained with good-quality samples are more meaningful than those from poor-quality samples. The clinician can be reassured that an appropriate patient sample was examined, due to cytological confirmation of the presence of sputum. The QC results for SCA showed agreement of 90%, which was lower than the 99% seen for AFB sensitivity on both direct and sediment smears. This result was expected, since SCA is less objective than observing the presence or absence of AFB. However, 90% agreement indicates that the results are highly reproducible.

The patient positivity rate with direct microscopy without SCA was 43.2%. This rate is considerably higher than the recommended positivity rate of 5-20% [23] and implies that case detection was not optimal--i.e. that the suspects referred for testing were likely to be more strongly positive than in a standard patient population. This was probably because of a selection bias in the patients referred for TB diagnosis. The hospital functioned as an unofficial referral hospital for people living with HIV/AIDS, and very sick patients often self-referred from all over the country. These patients could have had advanced TB infections. A larger incremental yield for bleach sedimentation and SCA might have been observed if the positivity rate was lower, and patients had been in less advanced stages of infection.

The main limitation of our results is that, due to a lack of TB culture facilities, we were unable to incorporate the gold standard of TB diagnosis: culture on Lowenstein-Jensen medium. We are therefore unable to describe the techniques with regard to increased sensitivity and potential false smear positive results, but only with regard to increased detection rate of AFB or incremental yield. Lack of culture confirmation could have resulted in false detection of positive results, which might have contributed to the incremental yield following sedimentation. The sample size was not reached despite extending the study to 18 months; unfortunately, logistical limitations prevented extending the study further. Bleach sedimentation with a case definition of two positive smears might have significantly increased yield if the required sample size had been reached. However, most results reached significance despite the lower-than-desired number of participants.

Mindouli hospital functioned as a comprehensive HIV diagnostic and treatment centre, and a large proportion of those screened for TB infection were HIV positive. Although it would have been interesting to have stratified the results based on HIV status, this was not done due to concerns about patient confidentiality if TB results were linked with HIV results at the laboratory. We are therefore unable to provide an estimate for the proportion of HIV TB coinfected patients.

Our loss-to-follow-up rate was low (3.2%), possibly because of emphasis on patient education during enrolment and provision of accommodation during the 3-day sample collection procedure, necessary since many patients came from outside the Mindouli area. Under routine conditions, without the provision of accommodation, a higher proportion of patients might be lost to follow up. The overnight delay in results following sedimentation could also lead to a higher patient loss to follow up. The loss to follow up rate after routine implementation should be monitored.

Due to the disadvantages associated with the bleach concentration technique (i.e. fragility of sediment smears, delayed results, and increased workload), routine implementation of the technique should only be considered after the feasibility of introduction in a particular context has been assessed, preferably following a pilot implementation phase.

## Conclusion

Implementation of bleach sedimentation will lead to a significant increase in the diagnosis of smear-positive TB patients. Implementation of SCA did not result in significantly increased diagnosis of TB. Efforts should be made to obtain good-quality samples, since bleach sedimentation of poor-quality samples is unlikely to result in a large incremental yield. With a case definition of one positive smear, we recommend that bleach sedimentation is implemented in settings where work has been done to improve sputum collection practices. Sample quality can be confirmed with SCA. A pilot study should be undertaken prior to routine implementation of bleach sedimentation to determine whether the technique will be of sufficient clinical benefit in a particular setting.

## Competing interests

The authors declare that they have no competing interests.

## Authors' contributions

PH conceived and carried out the study and wrote the first draft of the paper. PN was responsible for study implementation and protocol revision and commented on the draft. JG and MB performed data analysis and commented on the draft. VS designed the study, wrote the protocol, and commented on the draft. All authors read and approved the final manuscript.

## Pre-publication history

The pre-publication history for this paper can be accessed here:

http://www.biomedcentral.com/1471-2334/10/276/prepub
